# Supplementary material for: Using adversarial networks to extend brain computer interface decoding accuracy over time
Source: eLife. 2023 Aug 23;12:e84296. doi: 10.7554/eLife.84296 (PMC10446822; doi:10.7554/eLife.84296)
Supplement: Figure 2—source data 1. [file elife-84296-fig2-data1.docx]

**Figure 2 – source data 1.** Information for each dataset.

| Task* | Monkey | Date of birth | Cortical implant site | Cortical implant date | Number of recording sessions | Range of days | Range since array implantation | Motor outputs being recorded ** |
| --- | --- | --- | --- | --- | --- | --- | --- | --- |
| Isometric wrist | J | 2008-03-22 | Right M1 (hand area) | 2013-09-10 | 20 | 95 | 688 - 783 | EMGs from 7 muscles in left arm (ECU, FCU, ECRl, EDC, FCR, FDP, ECRb) |
|  | S | 2006-05-10 | Right M1 (hand area) | 2012-05-07 | 18 | 83 | 106 - 189 | EMGs from 8 muscles in left arm and hand (FDP1, FDP2, FCR, 1DI, FPB, EDC, MD, ECRl) |
| Grasping*** | G | 2008-11-01 | Right M1 (hand area) | 2019-07-23 | 8 | 53 | 23 - 76 | EMGs from 8 muscles in left arm and hand (FCR, FDP, PT, FPB, 1DI, SUP, ECU, EDC) |
|  | P | 2010-06-10 | Right M1 (hand area) | 2021-05-19 | 9 | 51 | 14 - 65 | EMGs from 11 muscles in left arm and hand (APB, FPB, Lum, PT, FDS, FDP, 1DI, 4DI, EPL, ECRl, EDC) |
| Center-out reach | C | 2008-03-29 | Left M1 (arm area) | 2016-08-09 | 12 | 38 | 49 - 87 | Hand velocities (v_x_, v_y_) |
|  | M | 2008-03-29 | Right M1 (arm area) | 2013-06-06 | 11 | 32 | 242 - 274 | Hand velocities (v_x_, v_y_) |
| Random-target reach | M | 2008-03-29 | Right M1 (arm area) | 2013-06-06 | 11 | 79 | 184 - 263 | Hand velocities (v_x_, v_y_) |

* For grasping, center-out, and random-target reach we tested all the available recording sessions as day-0, while for the isometric wrist task we tested 9 out of the available 20 sessions for monkey J and 8 out of the available 18 for monkey S. Each day in a dataset other than the designated day-0 was treated as a day-k, whether it occurred before or after day-0.

** Abbreviations for the muscles: ECU (extensor carpi ulnaris), FCU (flexor carpi ulnaris), ECRl (extensor carpi radialis longus), EDC (extensor digitorum communis), FCR (flexor carpi radialis), FDP (flexor digitorum profundus), ECRb (extensor carpi radialis brevis), FCR (flexor carpi radialis), 1DI (first dorsal interosseous), FPB (flexor pollicis brevis), MD (opponens digiti minimi), PT (pronator), SUP (supinator), APB (abductor pollicis brevis), Lum (lumbrical), FDS (flexor digitorum superficialis), 4DI (fourth dorsal interosseous), EPL (extensor pollicis longus).

*** Monkey G was trained to do key grasping, and Monkey P do power grasping.
